# Clark County Medical Society

**Published:** 1867-08

**Authors:** F. R. Payne, J. D. Mitchell

**Affiliations:** President; Secretary


### CLARK COUNTY MEDICAL SOCIETY.

This Society met at Marshall, July 1, 1867, eighteen members being present.

Good feeling prevailed throughout the meeting, and all seemed to be fully imbued with a spirit of inquiry and medical research.

After the business of the Society was transacted, Dr. J. C. Price read an article on the preparation and use of styptic colloid. It forms an excellent application in all abraded surfaces. He spoke of its good effects in Whitlow, after the use of the knife. He also read a paper on the "True Physician," in which he urged, with great force, the necessity of united action on the part of medical men against the inroads of impostors and quack nostrums. He spoke against the recent efforts on the part of speculators in human life to revive the old follies of magic incantation and *hocus pocus* in the form of "Snap Doctors," and other impositions.

Dr. Daniel Gard read a lengthy article on "Chronic Conjunctivitis," which was listened to with much interest. The Doctor has had a great deal of experience in the management of this disease, and his chief remedy is nitrate of silver, as a local application, aided by proper constitutional remedies. He uses the nitrate in the strength of from one to fifteen grains per ounce of Rose water.

On motion of Dr. Payne, Dr. Gard was requested to furnish a copy of his paper for publication in the CHICAGO MEDICAL EXAMINER.

Dr. Spencer remarked, that at our next meeting he would read a paper on the Nature and Treatment of Carcinomatous Tumors, and cancer in general.

Dr. Jennings offered the following resolution, which was unanimously adopted:

*Resolved*, That every member of this Society, at each regular meeting, be absolutely required to read an Essay on some medical subject, or report a case and treatment.

Dr. A. A. Lodge was unanimously elected a member of this Society.

Dr. Payne read a paper on the "Vis Conservatrix Nature." He impersonated this restorative power of nature, and contended that the difference between a good physician and no physician at all was not half so great as the difference between a good and a bad physician. At an early day this power was the *archeus* of Van Helmont, and the *animi* of Stahl. The Doctor remarked that this curative power of nature was strictly scientific, yet it was unfortunately and necessarily the main prop and back bone of all false theories and medical impostors. Were it not for this power the infinitesimal doses of the homœopath would no longer delude and swindle mankind. This paper was received, and ordered published.

Dr. Jennings gave the *post mortem* appearance of Mr. J. Stover, nineteen years old. This young man was examined by the members of this Society last March, and his disease was pronounced hyperthropy of the heart and hydrothorax. After death his heart was found greatly enlarged, walls thin, and about one gallon of water in the thorax. The treatment recommended last March did not seem to produce the least benefit. The disease had advanced beyond the reach of remedial agents. This case was fully discussed by Drs. Jennings, Lodge, Mitchell, Gard, and Goodell.

Dr. F. R. Payne presented an Essay on Cerebro-Spinal Meningitis, in which he reported two recent malignant cases, and gave the *post mortem* appearanca of one. This paper was ordered to be sent to the CHICAGO MEDICAL EXAMINER, for publication.

The Society then adjourned to meet in Martinsville on the first Wednesday in November next.

F. R. PAYNE, President.

J. D. MITCHELL, Secretary.

